# 3D-ALMOND-QSAR Models to Predict the Antidepressant Effect of Some Natural Compounds

**DOI:** 10.3390/pharmaceutics13091449

**Published:** 2021-09-10

**Authors:** Speranta Avram, Miruna Silvia Stan, Ana Maria Udrea, Cătălin Buiu, Anca Andreea Boboc, Maria Mernea

**Affiliations:** 1 Department of Anatomy, Animal Physiology and Biophysics, Faculty of Biology, University of Bucharest, SplaiulIndependentei, No 91-95, 050095 Bucharest, Romania; speranta.avram@gmail.com (S.A.); miruna.stan@bio.unibuc.ro (M.S.S.); maria.mernea@bio.unibuc.ro (M.M.); 2 Research Institute of the University of Bucharest–ICUB, University of Bucharest, 91–95, SplaiulIndependentei, 050095 Bucharest, Romania; ana.udrea@inflpr.ro; 3 Laser Department, National Institute for Laser, Plasma and Radiation Physics, 077125 Magurele, Romania; 4 Department of Automatic Control and Systems Engineering, Politehnica University of Bucharest, 313 SplaiulIndependenţei, 060042 Bucharest, Romania; 5 “Maria Sklodowska Curie” Emergency Children’s Hospital, 20, Constantin Brancoveanu Bd., 077120 Bucharest, Romania; anca.orzan@gmail.com; 6 Department of Pediatrics 8, “Carol Davila” University of Medicine and Pharmacy, EroiiSanitari Bd., 020021 Bucharest, Romania

**Keywords:** antidepressant, natural compounds, QSAR, molecular docking

## Abstract

The current treatment of depression involves antidepressant synthetic drugs that have a variety of side effects. In searching for alternatives, natural compounds could represent a solution, as many studies reported that such compounds modulate the nervous system and exhibit antidepressant effects. We used bioinformatics methods to predict the antidepressant effect of ten natural compounds with neuroleptic activity, reported in the literature. For all compounds we computed their drug-likeness, absorption, distribution, metabolism, excretion (ADME), and toxicity profiles. Their antidepressant and neuroleptic activities were predicted by 3D-ALMOND-QSAR models built by considering three important targets, namely serotonin transporter (SERT), 5-hydroxytryptamine receptor 1A (5-HT1A), and dopamine D2 receptor. For our QSAR models we have used the following molecular descriptors: hydrophobicity, electrostatic, and hydrogen bond donor/acceptor. Our results showed that all compounds present drug-likeness features as well as promising ADME features and no toxicity. Most compounds appear to modulate SERT, and fewer appear as ligands for 5-HT1A and D2 receptors. From our prediction, linalyl acetate appears as the only ligand for all three targets, neryl acetate appears as a ligand for SERT and D2 receptors, while 1,8-cineole appears as a ligand for 5-HT1A and D2 receptors.

## 1. Introduction

Depression is a common mental disorder, 264 million persons being affected worldwide, according to the WHO. A severe consequence of depression is suicide, and near 800,000 people commit suicide every year [1]. Depression can be treated by psychotherapy and medication involving antidepressant and antipsychotic drugs. Although these drugs have beneficial effects in the management of depression, their usage could lead to severe side effects like hepatotoxicity, weight gain, sexual dysfunction, cardiovascular disorders, central nervous system disturbances, etc. [2].

Natural compounds may represent a viable alternative in depression treatment with possibly fewer side effects, supporting their administration even in patients with comorbidities [3,4]. Here we used an in silico approach to predict the antidepressant activity of the following natural compounds: resveratrol, quercetin, limonene, sabinene, 1,8-cineole, chamazulene, linalyl acetate, germacrene D, nerol, and neryl acetate.

Resveratrol is a polyphenol with benefits in inflammation, brain diseases, and depression [4]. A study on irritable bowel syndrome rat model shows that resveratrol had inhibitory activity on the 5-hydroxytryptamine receptor 1A (5-HT1A), thus improving the brain–gut axis [5]. A review of twenty-two studies concludes that resveratrol has positive effects on animal models with depression, comparable with those of antidepressant drugs. Regarding safety, the same review concludes that resveratrol has an exceptional safety profile and only a few side effects [6].

Quercetin is a flavonoid whose antidepressant activity was studied on diabetic mice and compared with antidepressants fluoxetine and imipramine. Results show that quercetin had similar results with those drugs in diabetic mice but not in naive mice [7]. Another study on mice concludes that pre-administrated quercetin decreases stress-induced behaviour, regulates cholinergic and serotoninergic functions, has an anxiolytic and antidepressant effect, and boosts memory function [8]. Quercetin also inhibits the behavioral effects induced by corticotropin-releasing factor (anxiety and depression) in mice model study [9].

Limonene is a monocyclic monoterpene known for its antiviral, antibacterial, anticancer, and anti-inflammatory activities [10]. This compound also shows neuroprotective effects in a *Drosophila* model [11] and antidepressant-like activity (mediated by its anti-neuroinflammatory action and by lowering hippocampal nitrite levels) in a mice model [12]. Studies on mice models showed that limonene regulates dopamine levels and 5-HT function [13] and increases dopamine and norepinephrine levels [14]. 

Sabinene is a monoterpene with antimicrobial and antifungal activity, with possible effects on the central nervous system [15,16]. Sabinene is a component of *Origanum vulgare* (oregano) essential oil (4.95%), and it may show antidepressant-like activity in a rat model [17].

Eucalyptol, 1,8-cineole, is a monoterpenoid with benefits as an anti-mucolytic or anti-spasmolytic [18]. Additionally, 1,8-cineole inhalation shows an anxiolytic effect in both mice and humans [19,20]. Eucalyptol is found in various plants, including *Rosmarinus officinalis* (rosemary) aerial plants oil (8.58%). This oil shows inhibitory activity on 5-HT1A [21]. Even if this compound had antidepressant effects, the mechanism of action is not clear yet [14]. 

Chamazulene is an aromatic compound found in *Matricaria chamomilla* (chamomile) and is known as a compound with antioxidant, anti-inflammatory, and hepatoprotective effects [22]. Chamomile is known for its benefits in generalized anxiety and insomnia [23].

The monoterpene linalyl acetate is the main constituent of *Lavandula angustifolia* (lavander) essential oil. Lavender essential oil is known for its antidepressant and anxiolytic properties [24,25]. Linalyl acetate’s mechanism of action as an antidepressant is well studied: this compound has binding affinity for N-methyl-D-aspartate (NMDA) receptor [26], and reduces the activity of 5-HT1A receptor [24], but not of serotonin transporter (SERT) [26]. Nerol monoterpene can also be found in *Lavandula angustifolia* essential oil [27] or in *Rosa damascene* (damask rose) oil. It can decrease the lipid peroxidation levels, a process occurring under chronic mild stress [28].

Germacrene D is a sesquiterpenoid found in *Anthriscus nemorosa* (chervil) essential oil in a proportion of 5.6%. Studies on rats revealed that scopolamine-induced memory impairment, anxiety, and depression can be improved using chervil essential oil [14]. Neryl acetate is one of the main constituents of *Cananga odorata* (ilang-ilang) essential oil. This oil decreases dopamine levels and increases serotonin levels in mice [29].

The compounds that we have chosen to analyze are indexed in FooDB, a resource on food constituents, comprising detailed descriptions of compound, including their physiological and presumed health effects [30]. Except for sabiene, linalyl acetate, nerol, and neryl acetate, the compounds are also indexed in DrugBank, a database comprising extensive information on drugs and drugs targets, including natural compounds and herbs used in therapeutic products [31]. The applications of selected compounds as indexed in the two databases are summarized in Table 1. It is essential to highlight that DrugBank—or any other database, for that matter—does not index the considered compounds as antidepressants. Taking into account their promising benefits in depression, as revealed mostly by studies conducted in animal models, we considered here the possibility to reposition some of these compounds as antidepressants and even as neuroleptics to be used in the treatment of humans. 

Previously, we used structure-activity relationship (SAR) models to investigate the potential of natural compounds from *Mentha spicata* essential oil to modulate acetylcholinesterase and NMDA receptor, two important targets considered in Alzheimer’s disease therapy [32]. A similar approach can be applied in the case of other nervous system diseases, like depression. In depression, important therapy targets are SERT, dopamine receptor 2 (D2), and 5-HT1A [33,34]. In our previous work, we built quantitative structure-activity relationship (QSAR) models to predict the effect of candidate compounds against these targets [33,34]. 

Particularly useful in drug design, discovery, and development is 3D-QSAR methodology, which helps to understand the relationship between spatial parameters of molecules and their biological properties [35]. Recent studies have used 3D-QSAR as a screening step in drug repositioning strategies, some examples being [36,37,38,39]. In these cases, QSAR models were used to screen compounds from: (i) DrugBank database [31] in order to identify promising candidates that modulate histone deacetylases [37] or inhibit SARS-CoV main protease [38]; (ii) FDA approved drugs from ZINC database [40] that inhibit Sirt2 [39]; or (iii) FDA approved drugs from e-Drug3D database [41] to identify druggable compounds for iatrogenic botulism treatment [36]. 3D-QSAR is valuable in drug repositioning, being complementary to other methods like molecular docking, because it predicts the activity of compounds (high/low activity) and yields the molecular features important for their effect [39].

In the present study we built three QSAR models to screen our collection of natural compounds identified from the literature against SERT, D2, and 5-HT1A receptor in order to identify the most promising compound with antidepressant and neuroleptic activity. Additionally, we analyzed the interactions between receptors and lead ligands using molecular docking.

## 2. Materials and Methods

### 2.1. Preparation of Natural Compounds Structures

The present study looked at ten natural compounds, including resveratrol, quercetin, limonene, sabinene, 1,8-cineole, chamazulene, linalyl acetate, germacrene D, nerol, and neryl acetate, based on their potential antidepressant effects identified in the literature, as described in the Section 1. The antidepressant efficacy of these drugs is determined using SERT and 5-HT1A receptors, as well as the neuroleptic effect on the D2 receptor.

MOE software was used to model and optimize the 3D structures of molecules. We minimized the energy using the MMFF94x force field at a 0.005 gradient and Gasteiger-type charges [42].

### 2.2. Prediction of Compounds Drug- and Lead-Likeness Features

The Lipinski [43], Veber [44], Ghose [45], and Egan [46] filters, which were predicted in the SwissADME online tool [47], were used to evaluate the drug-likeness of the natural compounds. The analyzed compounds should not violate more than three of these rules. According to the Lipinski criteria, compounds must have a molecular weight lower than 500 Daltons, no more than 10 hydrogen bond acceptors, no more than 5 hydrogen bond donors, and a log octanol/water (Log P(o/w)) lower than 5. 

The molecular weight must be between 160 and 480, the Log P(o/w) must be between 0.4 and 5.6, the molar refractivity must be between 40 and 130, and the number of atoms must be between 20 and 70, according to the Ghose filter. 

If the total polar surface area is less than 140 and the number of rotatable bonds is less than 10, the Veber rule applies. The Log P(o/w) must be less than 5.88, and the total polar surface area must be less than 131, according to the Egan filter.

### 2.3. Computational Pharmacokinetics and Pharmacogenomics Profiles of Natural Compounds

The SMILES files of natural compounds were used to predict their absorption, distribution, metabolism, excretion, and toxicity (ADMET) profiles using the pkCSM database [48].

We started by calculating all of the ADMET entries that the bioinformatics portal sent us. The following were chosen as related to our research: (i) intestinal absorption (percentage)—a molecule with an absorption rate of less than 30% is deemed poorly absorbed; (ii) permeability of the blood-brain barrier (BBB) represented as log BBB (logarithm of the brain to plasma drug concentration ratio)—higher than 0.3 indicates high BBB permeability, while lower than 1 indicates low BBB permeability; (iii) central nervous system (CNS) permeability—a compound with a permeability-surface area product (logPS) higher than −2 can penetrate the CNS; (iv) fraction unbound (human) is represented by the ratio of the unbounded compound on plasmatic proteins; (v) substrate of renal organic cation transporter 2 (OCT2), the main renal uptake transporter that is expressed on the basolateral side of the proximal tubule.

We investigated the potential of the compounds to serve as inhibitors or substrates for cytochromes involved in the metabolization of neuropsychiatric medications, such as CYP2D6, CYP3A4, CYP1A2, CYP2C19, and CYP2C9, to predict their pharmacogenomic profile. The prediction of toxicity was a significant aspect of our research. 

We assessed AMES toxicity, hepatotoxicity, LD50 (median lethal dose), and maximum tolerated dose (human).

### 2.4. Building 3D-ALMOND-QSAR to Predict Natural Compounds Effects 

We used Pentacle software to create three 3D-QSAR-ALMOND models to predict the action of natural compounds on SERT, D2, and 5-HT1A receptors [33,49]. The three models are further called QSAR-SERT, QSAR-D2, and QSAR-5-HT1A in correlation with the target they address. 

For each molecule, we computed several molecular descriptors. Each descriptor’s contribution to biological activity was assessed singly or in groups of different combinations. The hydrophobicity, electrostatic, and hydrogen bond donor/acceptor features were found to be the most important statistical combination of molecular descriptors for all QSAR models.

The chemometric analysis was made using the regression analysis, partial least squares (PLS) within PENTACLE. The number of PLS components (latent variables, LVs = 5) was chosen to achieve optimum values of statistical parameters. These are r^2^ > 0.8 (fitted correlation coefficient) and q^2^ > 0.6 (cross-validated correlation coefficient). Additionally, SDEP (standard deviation of error prediction) and SDEC (standard deviation of error calculation) were evaluated. 

The generation of consistent statistical models depends on the quality of training, validation, and testing sets in terms of structural diversity and property values distribution.

QSAR-SERT model has amitriptyline, citalopram, clomipramine, desipramine, doxepin, escitalopram, fluoxetine, imipramine, lofepramine, paroxetine, sertraline, trazodone, venlafaxine, aripiprazole, chlorpromazine, clozapine, fluphenazine, haloperidol, risperidone, sertindole, and zotepine in the training set and, in the validation set, bupropion, olanzapine, quetiapine, thioridazine, ziprasidone, and fluvoxamine.

QSAR-5-HT1A model has amitriptyline, desipramine, doxepin, escitalopram, fluoxetine, trazodone, aripiprazole, chlorpromazine, fluphenazine, haloperidol, iloperidone, loxapine, olanzapine, prochlorperazine, quetiapine, risperidone, spiperone, trifluoperazine, and ziprasidone in the training set and, in the validation set, clozapine, sertindole, thioridazine, and zotepine.

QSAR-D2 model has amitriptyline, despyramine, flufenazine, haloperidol, iloperidone, loxapine, prochlorperazine, risperidone, spiperone, trifluoperazine, clomipramine, clozapine, mesoridazine, olanzapine, promazine, remoxipride, sertindole, thioridazine, and zotepine in the training set and, in the validation set, doxepine, aripriprazole, quetiapine, chlorpromazine, and ziprasidone. 

The test set used in all QSAR models is represented by the ten natural compounds that we investigated.

The biological activities of compounds in training and validation sets expressed as Ki values (inhibition constants) were retrieved from PDSP Ki Database—Psychoactive Drug Screening Program [50]. The three models that we built predict the biological activities of compounds as pKi values (log 1/Ki) for a better statistical analysis.

### 2.5. Molecular Docking Protocol

The interactions of the lead compounds identified using our QSAR models with SERT, D2, and 5-HT1A receptors were predicted using molecular docking.

The 3D protein structures were imported from Protein Data Bank in the case of SERT (PDB ID: 6VRH [51]) and D2 (PDB ID:6CM4 [52]) receptors; the structure of the 5-HT1A receptor was imported from AlphaFold [53].

The molecular docking was performed using the CDOCKER algorithm [54] implemented in Biovia Discovery Studio v16.1.0.15350 (BIOVIA Dassault Systemes, San Diego, CA, USA).

The ligands were docked in the drug binding cavities according to the PDB files used [51,52]. In the case of 5-HT1A, the binding site was identified by similarity with the site of the D2 receptor [52]. The Docking protocol was applied as described in the study of Rao et al. [55].

## 3. Results

### 3.1. Drug-Likeness, Pharmacokinetics, and Pharmacogenomics Profiles of Compounds

The structures of compounds were retrieved from PubChem database [56] as SMILES (Simplified Molecular Input Line Entry) files, as presented in Table 2. 

To determine the drug-likeness of compounds, we applied different filters, as presented in Table 2. As can be seen, all compounds comply with Lipinski, Veber, and Egan rules. In the case of the Ghose rule, only 1,8-cineole, limonene, and sabiene present one violation of the rule. These results show that the compounds present drug-likeness features and could present a good bioavailability.

ADME and toxicity profiles of compounds were computed, with an emphasis on human intestinal absorption (HIA), BBB and CNS permeabilities, human fraction unbound (HFU), renal OCT2 substrate, mutagenesis features-AMES, hepatotoxicity, maximum of tolerated dose (human), and LD50 (Table 3). 

Here, we also computed the biological activities of considered natural compounds at some very important human cytochromes involved in neuropsychiatric disorders, namely CYP2D6, CYP3A4, CYP1A2, CYP2C19, and CYP2C9 [57].

The biological activities of natural compounds were expressed as inhibitors or substrates of human cytochromes, as presented in Table 4. 

We intended to generate a pharmacogenomic pathway of natural compounds through these predictions, establishing if these are metabolized by the same cytochromes as classical antidepressants or neuroleptics, which is relevant when a combinatorial therapy involving classical antidepressants, classical neuroleptics, and natural compounds is indicated.

### 3.2. Natural Compounds’ Antidepressant Activities Predicted by 3D-ALMOND-QSAR

Three QSAR models (QSAR-SERT, QSAR-D2, and QSAR-5-HT1A) were built to predict the biological effect of natural compounds against SERT, 5-HT1A, and D2 receptors. In building the models we initially considered individual descriptors like hydrophobicity, hydrogen bond donor/acceptor, electrostatic, or steric. These models could not predict biological activities in correlation with experimental activities.

Further, we considered the contribution of several descriptors at the same time, which led to a significant improvement of the prediction accuracy of our models (r^2^ > 0.9, q^2^ > 0.8, SDEP < 0.5), the statistical parameters being given in Table 5.

The predicted activity of classical neuropsychiatric drugs in the training and validation sets was calculated according to the QSAR equations previously generated and was compared with experimental activity on SERT, 5-HT1A, and D2 receptors (Table 6). 

Our QSAR models are described by very good statistical parameters, which allow us to predict the biological activities of 1,8-cineole, limonene, sabinene, resveratrol, chamazulene, germacrene D, linalyl acetate, nerol, neryl acetate, and quercetin at SERT, 5-HT1A, and D2 by following the QSAR equation generated in ALMOND-Pentacle (Table 6).

**Figure 1:**
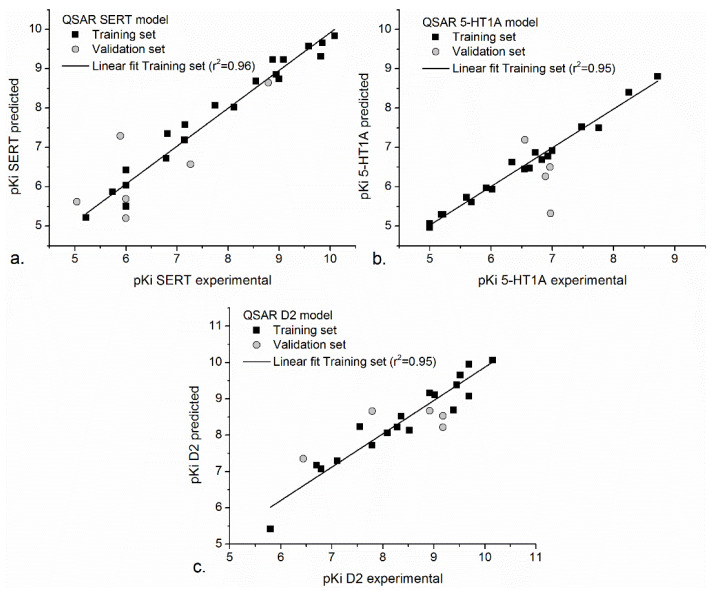
The correlation between experimental and predicted values of QSAR SERT (**a**), QSAR 5-HT1A (**b**), and QSAR D2 (**c**) models. Data were plotted and fitted using Origin Pro, version 9.2 (2015), OriginLab Corporation, Northampton, MA, USA.

The correlation between the training and validation sets of our QSAR models is also represented in Figure 1.

### 3.3. Molecular Docking 

The interaction of the most promising compounds acting on the three protein targets were investigated by molecular docking. Therefore, we docked linalyl acetate at SERT, D2, and 5-HT1A; neryl acetate was docked at SERT and 5-HT1A; and 1,8-cineole was docked at D2 and 5-HT1A (see Section 4.3). The binding of ligands was evaluated based on CDOCKER energy and CDOCKER interaction energy; values are presented in Table 7. 

**Figure 2:**
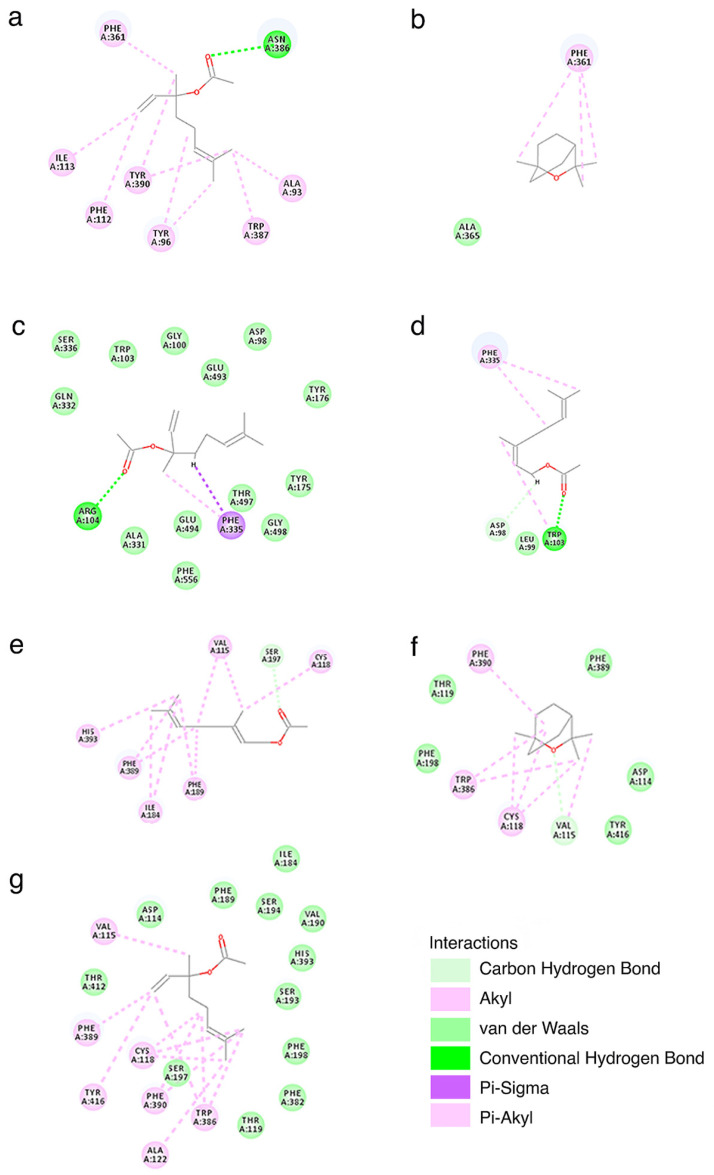
Interaction maps (2D) calculated based on the complexes formed by linalyl acetate with 5-HT1A (**a**), SERT (**c**), or D2 (**g**); 1,8-cineole with 5-HT1A (**b**) or D2 (**f**) and neryl acetate with SERT (**d**) or D2 (**e**). The maps were generated using Discovery Studio Visualizer v21.1.0.20298 (BIOVIA Dassault Systemes, San Diego, CA, USA).

We further analyzed the structural basis of the interaction between ligands and targets. The 2D interaction maps are presented in Figure 2.

## 4. Discussion

Drug repositioning involves the identification of novel treatments for diseases based on “old” drugs or compounds [58]. Several strategies were developed to achieve the identification of druggable compounds against novel targets, like QSAR studies, in conjunction with molecular docking [36], with molecular docking and molecular dynamics [37,38], and even with quantum mechanics/molecular mechanics methods [39]. High performance QSAR models can be obtained by using machine learning approaches to classify the molecular descriptors of compounds from large datasets [59]. Repositioning drug candidates can be identified using drug-drug interaction networks [60]; the method even allows the ranking of compounds into simple and complex multi-pathology therapies [61]. Unsupervised machine learning approaches can be used to establish dug–drug similarity networks based on drug–target interactions, which also lead to the identification of repositioning candidates [62]. Other approaches in drug repositioning, as well as limitations and recommendations, are presented in [58]. 

In the present study we performed a computational investigation on the possibility of repositioning some natural compounds as antidepressants and neuroleptics. Our tentative hypotheses were supported by previous experimental studies that report their antidepressant effects mainly in animals, as presented in the Section 1. Our strategy involved an initial filtering of compounds based on their drug-like properties, their predicted pharmacokinetic, and pharmacogenomic profiles (Section 4.1 and Section 4.2). In the following step, QSAR models were built to predict the most active inhibitors of three druggable targets in depression, namely SERT, 5-HT1A, and D2 receptors. We selected the potent compounds that modulate three or at least two targets at once (Section 4.3). The interactions between lead compounds and the targets were addressed by molecular docking (Section 4.4).

### 4.1. Assessment of Compounds Drug-Likeness Features

Generally, all the natural compounds studied here are in agreement with the medicinal chemistry rules (Table 2). One rule of the Ghose filter (MW < 160) was violated only by 1,8-cineole, limonene, and sabinene. The molecular descriptors of resveratrol, chamazulene, germacrene D, linalyl acetate, nerol, neryl acetate, and quercetin presented values within the ranges defined by Lipinski, Veber, Ghose, and Egan rules. Our results suggest that the natural compounds considered here present drug-likeness features and should be further characterized by computing their pharmacokinetic and pharmacodynamic profiles.

### 4.2. Computational Pharmacokinetics and Pharmacogenomics Profiles of Natural Compounds

The ADME profiles predicted for the compounds showed that the human intestinal absorption parameter lies in the 77.20% (quercitin) to 96.50% (1,8-cineole) range, as shown in Table 3. Very good human intestinal absorption values were recorded for 1,8-cineole, neryl acetate, and limonene.

Regarding the distribution of the natural compounds in the human body, the human fraction unbound parameter presented a large variation, from 0.18 (resveratrol) to 0.55 (1,8-cineole). Unfortunately, the selected natural compounds presented low human fraction unbound percents. Other critical parameters for describing the natural compounds’ distribution in the body are BBB and CNS permeabilities. As shown in Table 3, the considered natural compounds recorded very good BBB permeability values, log BBB ranging from 0.83 (sabinene) to −1.09 (quercitin). An easy BBB penetration was recorded for sabinene, limonene, chamazulene, germacrene D, and nerol, our results being in good agreement with the experimental studies [14,63]. The predicted CNS permeability values range from −1.46 (sabinene) to −3.06 (quercitin). These suggest that the considered natural compounds should have a good CNS permeability, the most permeable compounds being sabinene, chamazulene, resveratrol, germacrene D, and nerol (Table 3). These results are supported by experimental studies showing a possible activity of these compounds at CNS level [15].

The metabolism of compounds was addressed by predicting their affinities for human cytochrome P450 proteins (CYPs): CYP2D6, CYP3A4, CYP1A2, CYP2C19, and CYP2C9 (Table 4). Our results revealed that resveratrol inhibits CYP2D6, CYP3A4, CYP1A2, CYP2C19, and CYP2C9, quercitin and chamazulene inhibit CYP1A2. Our results are in agreement with an experimental study mentioning that resveratrol modified the metabolism of aripiprazole by CYP2D6 and CYP3A4 [64]. The predicted inhibitory activity of quercetin on CYP1A2 is supported by a previous study [65] showing that quercetin is able to change the metabolism of melatonin by CYP1A2. Quercetin was also reported to be a strong inhibitor of CYP2D6 and a moderate inhibitor of CYP3A4 [66]. Chamazulene was experimentally proved to be a potent inhibitor of CYP1A2, CYP4A4, and CYP2D6 [67].

Important results were recorded for the elimination rate: none of the ten natural compounds are renal OCT2 substrates. In our study, high importance was given to predicting the toxicity of compounds. Our results predict that none of the compounds should present hepatotoxicity, cardiotoxicity, or AMES features. Additionally, we evaluated the maximum tolerated dose (human) and LD50 of natural compounds. Predicted LD50 values vary in a short range, from 1.54 (sabinene) to 2.47 (quercetin). A significant fluctuation was recorded for the maximum tolerated dose, from 0.05 mol/Kg (chamazulene) to 0.85 mol/Kg (nerol). Taken together, our results suggest that none of the natural compounds that we considered are toxic.

### 4.3. Predicted Pharmacodynamic Profiles of Natural Compounds on SERT, 5H-T1A, and D2 Active Sites by 3D-ALMOND-QSAR

The application of the QSAR-SERT model to molecules from the training and testing sets resulted in a suitable correlation between experimental and calculated biological activities. The differences between experimental and predicted biological activities for the molecules in the training set vary from 0.00 (fluphenazine) and −0.53 (zotepine), while the differences between experimental and predicted biological activities for the molecules in the validation set vary from 0.15 (fluvoxamine) and −1.40 (thioridazine). The statistical parameters (Table 5) and the good correlation between experimental and predicted biological activities of classical neuropsychiatric drugs support the strong power of prediction of QSAR-SERT model. Therefore, the model was used to predict the biological activities of natural compounds against SERT. These are presented in Table 6. 

In order to evaluate the potency of natural compounds against SERT, their predicted biological effects were subtracted from the value obtained for paroxetine, the most active compound from the training set (pKi experimental = 10.09). The results show that natural compounds such as limonene (pKi_paroxetine_-pKi_limonene_ = −0.64), sabinene (pKi_paroxetine_-pKi_sabinene_ = −0.72), chamazulene (pKi_paroxetine_-pKi_chamazulene_ = −0.59), germacrene D (pKi_paroxetine_-pKi_germacrene D_ = −0.57), linalyl acetate (pKi_paroxetine_-pKi_linalyl acetate_ = −0.69), nerol (pKi_paroxetine_-pKi_nerol_ = −0.35), and neryl acetate (pKi_paroxetine_-pKi_neryl acetate_ = 0.52) have a strong antidepressant character. 

Our results are in accord with other studies [14,68] which mention that limonene and sabinene reduce the depression-related behaviors in a similar manner with fluoxetine [69], linalyl acetate increases 5-HT levels in the amygdala, hypothalamus, and hippocampus of mice [70] and nerol effectively reduces the symptoms of depression and sensitivity.

In QSAR-5-HT1A model, a good correlation between predicted and experimental biological activities was noticed for molecules from the training set, where residual value varies from 0.04 (fluoxetine, iloperidone) to −0.28 (amitriptyline) and also for the validation set, where the residual value varies from 0.46 (thioridazine) to 1.65 (clozapine). The biological activities of natural compounds at 5-HT1A were evaluated relative to the activity of ziprasidone, the most active compound of the training set (pKi experimental = 8.72). We noticed that the natural compounds presented a middle antidepressant activity. A good affinity was predicted in the case of 1,8-cineole (pKi_ziprasidone_-pKi_1,8-cineole_ = 1.83) and linalyl acetate (pKi_ziprasidone_-pKi_linalyl acetate_ = 1.83) (Table 6).

The neuroleptic activity of natural compounds was evaluated by their affinity at D2 receptor. The power of prediction of QSAR-D2 model was sustained by the good statistical parameters (Table 5). Similar to previous QSAR models, a good correlation between predicted and experimental biological activities were obtained in both training and validation sets (Table 6). The neuroleptic activity of natural compounds was evaluated versus spiperone (pKi experimental = 10.15) and we noticed that a good neuroleptic activity is recorded by quercetin (pKi_spiperone_-pKi_quercetin_ = −1.76), neryl acetate (pKi_spiperone_-pKi_neryl acetate_ = −2.01), linalyl acetate (pKi_spiperone_-pKi_linalyl acetate_ = −2.04), and 1,8-cineole (pKi_spiperone_-pKi_1,8-cineole_ = −2.04). The affinity of quercitin at D2 is close to the affinity of mesoridazine, loxapine, and olanzapine, our results being supported by experimental studies [71].

### 4.4. Molecular Basis of the Interaction between Lead Compounds and Targets

The docking scores that we calculated were CDOKER energy (calculated based on ligand strain energy and receptor-ligand interaction energy) and CDOCKER interaction energy (calculated based on ligand-receptor nonbonded interaction energy). In the case of linalyl acetate acting on the three targets, neryl acetate acting on SERT, and 5-HT1A and 1,8-cineole acting on D2 and 5-HT1A we obtained negative CDOCKER interaction energies, confirming that the ligands present favorable interaction energies with the targets (Table 7). The most favorable interaction energies were obtained for linalyl acetate and neryl acetate, while less favorable energies were calculated for 1,8-cinole.

By analyzing the 2D interaction maps presented in Figure 2 we observe that compounds form hydrogen bonds with the targets in the case of linalyl acetate with SERT and 5-HT1A or in the case of neryl acetate and SERT. Other types of interactions established by the compounds and the targets fall in the category of: (i) van der Waals interactions—important for the binding of linalyl acetate to SERT, linalyl acetate to D2, or 1,8-cineole to D2; (ii) alkyl or π-alkyl interactions—important for the binding of linalyl acetate to 5-HT1A or D2, neryl acetate to D2, or 1,8-cineole to D2; and (iii) µ-σ interactions—which appear only in the case of linalyl acetate binding to SERT. The types of interactions that we identified are consistent with the molecular properties relevant for target binding that we identified using our QSAR models.

## 5. Conclusions

Medication repositioning is a quick way to employ an existing drug to treat new diseases [72,73,74]. The present study has investigated the opportunity to reposition ten natural compounds identified from the literature, namely resveratrol, quercetin, limonene, sabinene, 1,8-cineole, chamazulene, linalyl acetate, germacrene D, nerol, and neryl acetate, as antidepressants and even as neuroleptics. These compounds are found in common fruits, spices, and tea herbs. All compounds are indexed in databases (DrugBank, FooDB) with different effects like anti-inflammatory, antioxidant, antimicrobial, antiallergic, or anti cancer, but none of them are indexed as antidepressants. Several experimental studies conducted mostly in animal models point toward their antidepressive effects. Therefore, we used computational methods to address the ability of compounds to modulate three major targets in depression, namely SERT, 5-HT1A, and D2 receptors and compared their predicted effect with the effect of potent drugs used in clinics. 

All ten compounds present drug-likeness features and no toxicity, meaning that they could be used in therapy. Their ADME features showed a very good intestinal absorption, as well as a good BBB and CNS permeability, suggesting that the compounds can reach the brain, where they should exert their biological effects.

Their biological activities relevant to depression were determined against SERT, 5-HT1A, and D2 receptors. For each target, we built powerful QSAR models that were trained and validated based on synthesis drugs that modulate their function. When predicting the effect of natural compounds, we determined that most compounds, namely limonene, sabiene, chamazulene, germacrene D, linalyl acetate, nerol, and neryl acetate, should inhibit SERT to an extent similar to paroxetine. Only two compounds appear as candidates to modulate 5-HT1A, namely 1,8-cineole and linalyl acetate, in a manner comparable with fluoxetine. Concerning the neuroleptic effect of compounds, quercetin, neryl acetate, linalyl acetate, and 1,8-cineole could be active against D2 receptors, in a similar manner with ziprasidone. Overall, we identified linalyl acetate as a strong affinity ligand for all three targets (SERT, 5-HT1A, and D2 receptor), and we consider it to be a promising antidepressant compound. Neryl acetate appeared as a promising ligand for both SERT and D2, while 1,8-cineole appears as a common ligand for 5-HT1A and D2 receptors. Molecular docking results confirm the favorable interaction between lead compounds and the targets.

The results obtained here show that linalyl acetate, neryl acetate, and 1,8-cineole target the proteins relevant in depression and present drug-likeness features, suitable ADME profiles, and no toxicity, suggesting they represent viable candidates for repurposing as antidepressants. Our simulation study offers evidence on the molecular mechanism of these compounds and the results should be confirmed experimentally. Results obtained here can be the starting point for studies on the repositioning of natural compounds and plants for an alternative treatment of depression, with significant efficiency, but reduced side effects, that can be administered even to patients with comorbidities or during pregnancy. 

## Figures and Tables

**Table 1:** Applications of the natural compounds indexed in the critical data bases DrugBank 5.1.8 [31] and Foodb [30].

Compound	DrugBank Accession Number	Medical Applications	Foodb Accession Number	Medical Applications
resveratrol	DB02709	anti-inflammatory, antioxidant, anticancer effects	FDB031212	suppresses NF-kappa B activation in HSV infected cells
quercetin	DB04216	specific quinone reductase 2 (QR2) inhibitor, may contribute to killing the malaria causing parasites	FDB011904	non-specific protein kinase enzyme inhibitor, agonist of the G protein-coupled estrogen receptor in human breast cancer cell lines
limonene	DB08921	common in cosmetic products, a flavoring to mask the bitter taste of alkaloids, a fragrance in perfumery	FDB013567	antimicrobial, expectorant
sabinene	not available	not available	FDB001456	not available
1,8-cineole	DB03852	controls airway mucus hypersecretion and asthma via anti-inflammatory cytokine inhibition, eucalyptol reduces inflammation and pain	FDB014616	antibronchitis, antiallergic
chamazulene	DB15931	not available	FDB015363	analgesic, antioxidant
linalyl acetate	not available	not available	FDB019133	antimicrobial, antioxidant, flavor
germacrene D	DB11276	as component of pine needle oil is used as disinfectant, lubricant, sanitizer, antimicrobial, insecticide	FDB003856	pesticide
nerol	not available	not available	FDB014945	antimicrobial, flavor, perfumery
neryl acetate	not available	not available	FDB013794	antimicrobial, flavor, perfumery

**Table 2:** The name of natural compounds and PubChem ID, 2D structure, SMILES [56], natural source, compound FooDB ID [30], and the druglikness features [47].

Compound		Smiles	Natural Source/FooDB Id	Lipinski	Veber	Ghose	Egan
1,8-cineole [PubChem ID = 2758]	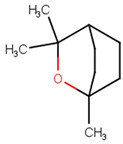	CC1(C2CCC(O1)(CC2)C)C	eucalyptus, sage [FDB014616]	YES	YES	No; 1 violation: MW < 160	YES
limonene [PubChem ID = 22311]	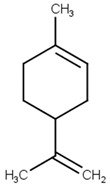	CC1=CCC(CC1)C(=C)C	peppermint, spearmint [FDB013567]	YES	YES	No; 1 violation: MW < 160	YES
sabinene [PubChem ID = 18818]	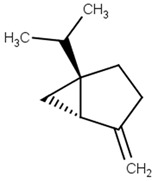	CC(C)C12CCC(=C)C1C2	lemon, mint [FDB001454]	YES	YES	No; 1 violation: MW < 160	YES
resveratrol [PubChem ID = 445154]	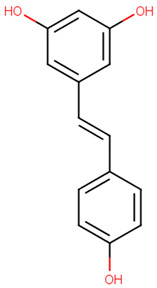	C1=CC(=CC=C1C=CC2=CC(=CC(=C2)O)O)O	skin of grapes [FDB031212]	YES	YES	YES	YES
chamazulene [PubChem ID = 10719]	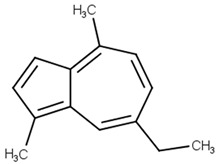	CCC1=CC2=C(C=CC2=C(C=C1)C)C	german chamomile, roman chamomile [FDB015363]	YES	YES	YES	YES
germacrene D [PubChem ID = 5317570]	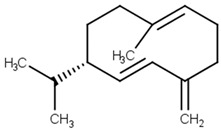	CC1=CCCC(=C)C=CC(CC1)C(C)C	Peppermint [FDB003856]	YES	YES	YES	YES
linalyl acetate [PubChem ID = 8294]	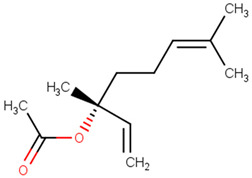	CC(=CCCC(C)(C=C)OC(=O)C)C	sage [FDB019133]	YES	YES	YES	YES
nerol [PubChem ID = 643820]	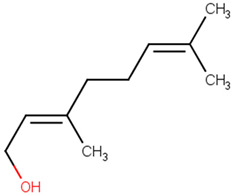	CC(=CCCC(=CCO)C)C	common grapes [FDB014945]	YES	YES	YES	YES
neryl acetate PubChem [ID = 1549025]	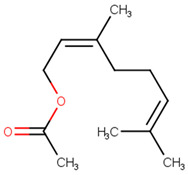	CC(=CCC/C(=C\COC(=O)C)/C)C	lemon balm, peppermint [FDB014946]	YES	YES	YES	YES
quercetin [PubChem ID = 5280343]	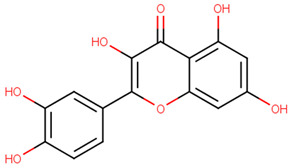	C1=CC(=C(C=C1C2=C(C(=O)C3=C(C=C(C=C3O2)O)O)O)O)O	Grape [FDB011904]	YES	YES	YES	YES

**Table 3:** Computed human intestinal absorption (HIA), blood-brain barrier permeability (log BBB), CNS permeability, human fraction unbound (HFU), maximum tolerated dose (human), and LD50 for selected compounds.

Compound	HIA	Log BBB	CNS Permeability	HFU	Max. Tolerated Dose (Human)	LD50
1,8-cineole	96.50	0.36	−2.97	0.55	0.55	2.01
limonene	95.89	0.72	−2.37	0.48	0.77	1.88
sabinene	95.35	0.83	−1.46	0.29	0.36	1.54
resveratrol	89.05	−0.04	−2.09	0.18	0.48	1.79
chamazulene	94.50	0.79	−1.82	0.24	0.05	1.45
germacrene D	95.59	0.72	−2.13	0.26	0.49	1.63
linalyl acetate	95.27	0.51	−2.37	0.42	0.54	1.72
nerol	93.46	0.62	−2.17	0.44	0.85	1.71
neryl acetate	96.06	0.56	−2.19	0.37	0.74	1.95
quercetin	77.20	−1.09	−3.06	0.20	0.49	2.47

**Table 4:** The inhibitor/substrate features of natural compounds at CYP2D6, CYP3A4, CYP1A2, CYP2C19 and CYP2C9.

Compound	CYP2D6 Substrate/ Inhibitor	CYP3A4 Substrate/ Inhibitor	CYP1A2 Inhibitor	CYP2C19 Inhibitor	CYP2C9 Inhibitor
1,8-cineole	no/no	no/no	no	no	no
limonene	no/no	no/no	no	no	no
sabinene	no/no	no/no	no	no	no
resveratrol	no/yes	no/yes	yes	yes	yes
chamazulene	no/no	no/no	no	no	no
germacrene d	no/no	no/no	no	no	no
linalyl acetate	no/no	no/no	no	no	no
nerol	no/no	no/no	no	no	no
neryl acetate	no/no	no/no	no	no	no
quercetin	no/no	no/no	yes	no	no

**Table 5:** Summary of the ALMOND statistical parameters in QSAR-SERT, QSAR-5-HT1A, and QSAR-D2.

Statistical Parameters	QSAR-SERT	QSAR-5HT-1A	QSAR-D2
No. of molecules in a training set	21	19	19
q^2^	0.80	0.90	0.83
r^2^	0.96	0.95	0.95
SDEP	0.50	0.29	0.40

**Table 6:** Predicted and experimental biological activities of compounds at SERT, 5-HT1A, and D2 receptors. The biological activities of molecules in the validation set are in italics. In brackets are the predicted biological activities of natural compounds versus the most active compounds of each QSAR model (paroxetine in QSAR-SERT; ziprasidone in QSAR-5HT-1A; spiperone in QSAR-D2).

Compounds	pKiSERT_exp_/ pKiSERT_predicted_	pKi 5-HT1A_exp_/ pKi 5-HT1A_predicted_	pKi D2_exp_/ pKi D2_predicted_
amitriptyline	8.55/8.68	6.34/6.62	6.70/7.17
citalopram	9.00/8.74	-	-
clomipramine	9.85/9.66	-	7.11/7.29
desipramine	7.75/8.07	5.19/5.29	5.80/5.42
doxepin	7.16/7.58	6.55/6.45	*6.44/7.35*
escitalopram	8.95/8.85	5.00/5.06	-
fluoxetine	9.09/9.23	5.00/4.96	-
imipramine	9.82/9.31	-	-
lofepramine	7.15/7.19	-	-
paroxetine	10.09/9.83	-	-
sertraline	9.58/9.57	-	-
trazodone	6.79/6.72	7.00/6.91	-
venlafaxine	8.12/8.02	-	-
aripiprazole	5.74/5.87	8.25/8.40	*9.18/8.53*
chlorpromazine	8.88/9.23	*6.93/6.78*	9.18/8.22
clozapine	6.00/5.49	*6.97/5.32*	7.55/8.23
fluphenazine	5.22/5.22	6.83/6.68	9.69/9.95
haloperidol	6.00/5.51	5.92/5.97	9.45/9.38
risperidone	6.00/6.04	6.72/6.87	9.52/9.65
sertindole	6.00/6.42	*6.55/7.19*	9.02/9.11
zotepine	6.82/7.35	*6.89/6.26*	8.09/8.06
bupropion	*5.04/5.62*	-	-
olanzapine	*6.00/5.69*	5.68/5.61	8.52/8.13
quetiapine,	*6.00/5.20*	6.63/6.47	*7.79/8.66*
thioridazine	*5.89/7.29*	*6.96/6.50*	9.39/8.69
ziprasidone	*7.27/6.57*	8.72/8.80	*8.92/8.67*
fluvoxamine	*8.79/8.64*	-	-
iloperidone	-	7.48/7.52	9.45/9.38
loxapine	-	5.60/5.73	8.28/8.22
prochlorperazine	-	5.22/5.30	9.69/9.07
spiperone	-	7.76/7.50	10.15/10.06
trifluoperidine	-	6.02/5.93	-
mesoridazine	-	-	8.36/8.52
promazine	-	-	6.79/7.07
remoxipride	-	-	7.79/7.72
trifluoperazine	-	-	8.92/9.16
Natural compounds			
1,8-cineole	8.57 (−1.52)	7.09 (−1.63)	8.11 (−2.04)
limonene	9.45 (−0.64)	6.73 (−1.99)	7.99 (−2.16)
sabinene	9.37 (−0.72)	6.68 (−2.04)	7.98 (−2.17)
resveratrol	8.68 (−1.41)	5.23 (−3.49)	6.72 (−3.43)
chamazulene	9.50 (−0.59)	6.51 (−2.21)	7.96 (−2.19)
germacrene D	9.52 (−0.57)	6.49 (−2.23)	7.90 (−2.25)
linalyl acetate	9.40 (−0.69)	6.89 (−1.83)	8.11 (−2.04)
nerol	9.74 (−0.35)	6.17 (−2.55)	8.05 (−2.10)
neryl acetate	10.61 (0.52)	6.06 (−2.66)	8.14 (−2.01)
quercetin	6.42 (−3.67)	5.87 (−2.85)	8.39 (−1.76)

**Table 7:** Molecular docking predictions of interactions between molecular targets in depression, natural compounds linalyl acetate, neryl acetate, and 1,8-cineole and CDOCKER scores calculated for analyzed ligands.

Compound	Target	-CDOCKER_ENERGY	-CDOCKER_INTERACTION_ENERGY
linalyl acetate	SERT	4.65	31.46
linalyl acetate	D2	−3.94	30.82
linalyl acetate	5-HT1A	−1.96	27.17
neryl acetate	SERT	−11.64	31.29
neryl acetate	5-HT1A	−9.79	33.85
1,8-cineole	D2	−14.66	17.25
1,8-cineole	5-HT1A	−8.22	19.07

## References

[1] World Health Organization (WHO) Depression. https://www.who.int/news-room/fact-sheets/detail/depression.

[2] Carvalho A.F., Sharma M.S., Brunoni A.R., Vieta E., Fava G.A. (2016). The Safety, Tolerability and Risks Associated with the Use of Newer Generation Antidepressant Drugs: A Critical Review of the Literature. Psychother. Psychosom..

[3] Yeung K.S., Hernandez M., Mao J.J., Haviland I., Gubili J. (2018). Herbal medicine for depression and anxiety: A systematic review with assessment of potential psycho-oncologic relevance. Phytother. Res..

[4] Udrea A.-M., Puia A., Shaposhnikov S., Avram S. (2018). Computational approaches of new perspectives in the treatment of depression during pregnancy. Target.

[5] Yu Y.-C., Li J., Zhang M., Pan J.-C., Yu Y., Zhang J.-B., Zheng L., Si J.-M., Xu Y. (2019). Resveratrol improves brain-gut axis by regulation of 5-HT-dependent signaling in the rat model of irritable bowel syndrome. Front. Cell. Neurosci..

[6] Moore A., Beidler J., Hong M.Y. (2018). Resveratrol and depression in animal models: A systematic review of the biological mechanisms. Molecules.

[7] Anjaneyulu M., Chopra K., Kaur I. (2003). Antidepressant Activity of Quercetin, a Bioflavonoid, in Streptozotocin-Induced Diabetic Mice. J. Med. Food.

[8] Samad N., Saleem A., Yasmin F., Shehzad M. (2018). Quercetin protects against stress-induced anxiety-and depression-like behavior and improves memory in male mice. Physiol. Res..

[9] Bhutada P., Mundhada Y., Bansod K., Ubgade A., Quazi M., Umathe S., Mundhada D. (2010). Reversal by quercetin of corticotrophin releasing factor induced anxiety- and depression-like effect in mice. Prog. Neuro-Psychopharmacol. Biol. Psychiatry.

[10] Mukhtar Y.M., Adu-Frimpong M., Xu X., Yu J. (2018). Biochemical significance of limonene and its metabolites: Future prospects for designing and developing highly potent anticancer drugs. Biosci. Rep..

[11] Shin M., Liu Q.F., Choi B., Shin C., Lee B., Yuan C., Song Y.J., Yun H.S., Lee I.-S., Koo B.-S. (2020). Neuroprotective effects of limonene (+) against Aβ42-induced neurotoxicity in a Drosophila model of Alzheimer’s disease. Biol. Pharm. Bull..

[12] Lorigooini Z., Boroujeni S.N., Sayyadi-Shahraki M., Rahimi-Madiseh M., Bijad E., Amini-Khoei H. (2021). Limonene through Attenuation of Neuroinflammation and Nitrite Level Exerts Antidepressant-Like Effect on Mouse Model of Maternal Separation Stress. Behav. Neurol..

[13] Yun J. (2014). Limonene inhibits methamphetamine-induced locomotor activity via regulation of 5-HT neuronal function and dopamine release. Phytomedicine.

[14] Zhang Y., Long Y., Yu S., Li D., Yang M., Guan Y., Zhang D., Wan J., Liu S., Shi A. (2020). Natural volatile oils derived from herbal medicines: A promising therapy way for treating depressive disorder. Pharmacol. Res..

[15] Caputo L., Nazzaro F., Souza L.F., Aliberti L., De Martino L., Fratianni F., Coppola R., De Feo V. (2017). Laurus nobilis: Composition of essential oil and its biological activities. Molecules.

[16] Fidan H., Stefanova G., Kostova I., Stankov S., Damyanova S., Stoyanova A., Zheljazkov V.D. (2019). Chemical composition and antimicrobial activity of Laurus nobilis L. essential oils from Bulgaria. Molecules.

[17] Amiresmaeili A., Roohollahi S., Mostafavi A., Askari N. (2018). Effects of oregano essential oil on brain TLR4 and TLR2 gene expression and depressive-like behavior in a rat model. Res. Pharm. Sci..

[18] Juergens U. (2014). Anti-inflammatory properties of the monoterpene 1.8-cineole: Current evidence for co-medication in inflammatory airway diseases. Drug Res..

[19] Kim K.Y., Seo H.J., Min S.S., Park M., Seol G.H. (2014). The effect of 1, 8-cineole inhalation on preoperative anxiety: A randomized clinical trial. Evid.-Based Complementary Altern. Med..

[20] Dougnon G., Ito M. (2020). Inhalation administration of the bicyclic ethers 1, 8-and 1, 4-cineole prevent anxiety and depressive-like behaviours in mice. Molecules.

[21] Martínez A.L., González-Trujano M.E., Pellicer F., López-Muñoz F.J., Navarrete A. (2009). Antinociceptive effect and GC/MS analysis of Rosmarinus officinalis L. essential oil from its aerial parts. Planta Med..

[22] Wang X., Dong K., Ma Y., Jin Q., Yin S., Wang S. (2020). Hepatoprotective effects of chamazulene against alcohol-induced liver damage by alleviation of oxidative stress in rat models. Open Life Sci..

[23] Mao J.J., Xie S.X., Keefe J.R., Soeller I., Li Q.S., Amsterdam J.D. (2016). Long-term chamomile (Matricaria chamomilla L.) treatment for generalized anxiety disorder: A randomized clinical trial. Phytomedicine.

[24] Malcolm B.J., Tallian K. (2017). Essential oil of lavender in anxiety disorders: Ready for prime time?. Ment. Health Clin..

[25] Donelli D., Antonelli M., Bellinazzi C., Gensini G.F., Firenzuoli F. (2019). Effects of lavender on anxiety: A systematic review and meta-analysis. Phytomedicine.

[26] López V., Nielsen B., Solas M., Ramírez M.J., Jäger A.K. (2017). Exploring pharmacological mechanisms of lavender (Lavandula angustifolia) essential oil on central nervous system targets. Front. Pharmacol..

[27] Saki K., Bahmani M., Rafieian-Kopaei M. (2014). The effect of most important medicinal plants on two importnt psychiatric disorders (anxiety and depression)-a review. Asian Pac. J. Trop. Med..

[28] Nazıroğlu M., Kozlu S., Yorgancıgil E., Uğuz A.C., Karakuş K. (2013). Rose oil (from Rosa × damascena Mill.) vapor attenuates depression-induced oxidative toxicity in rat brain. J. Nat. Med..

[29] Zhang N., Zhang L., Feng L., Yao L. (2016). The anxiolytic effect of essential oil of Cananga odorata exposure on mice and determination of its major active constituents. Phytomedicine.

[30] FooDB, Version 1.0. www.foodb.ca.

[31] Wishart D.S., Knox C., Guo A.C., Shrivastava S., Hassanali M., Stothard P., Chang Z., Woolsey J. (2006). DrugBank: A comprehensive resource for in silico drug discovery and exploration. Nucleic Acids Res..

[32] Avram S., Mernea M., Bagci E., Hritcu L., Borcan L.C., Mihailescu D.F. (2017). Advanced Structure-activity Relationships Applied to Mentha spicata L. Subsp. spicata Essential Oil Compounds as AChE and NMDA Ligands, in Comparison with Donepezil, Galantamine and Memantine—New Approach in Brain Disorders Pharmacology. CNS Neurol. Disord.-Drug Targets.

[33] Avram S., Duda-Seiman D., Borcan F., Wolschann P. (2011). QSAR-CoMSIA applied to antipsychotic drugs with their dopamine D2 and serotonine 5HT2A membrane receptors. J. Serb. Chem. Soc..

[34] Avram S., Buiu C., Duda-Seiman D.M., Duda-Seiman C., Mihailescu D. (2010). 3D-QSAR design of new escitalopram derivatives for the treatment of major depressive disorders. Sci. Pharm..

[35] Silverman R.B., Silverman R.B. (2004). Chapter 2-Drug Discovery, Design, and Development. The Organic Chemistry of Drug Design and Drug Action.

[36] Floresta G., Patamia V., Gentile D., Molteni F., Santamato A., Rescifina A., Vecchio M. (2020). Repurposing of FDA-Approved Drugs for Treating Iatrogenic Botulism: A Paired 3D-QSAR/Docking Approach. Chem. Med. Chem..

[37] Liu J., Zhu Y., He Y., Zhu H., Gao Y., Li Z., Zhu J., Sun X., Fang F., Wen H. (2020). Combined pharmacophore modeling, 3D-QSAR and docking studies to identify novel HDAC inhibitors using drug repurposing. J. Biomol. Struct. Dyn..

[38] Tejera E., Munteanu C.R., López-Cortés A., Cabrera-Andrade A., Pérez-Castillo Y. (2020). Drugs repurposing using QSAR, docking and molecular dynamics for possible inhibitors of the SARS-CoV-2 Mpro protease. Molecules.

[39] Bharadwaj S., Dubey A., Kamboj N.K., Sahoo A.K., Kang S.G., Yadava U. (2021). Drug repurposing for ligand-induced rearrangement of Sirt2 active site-based inhibitors via molecular modeling and quantum mechanics calculations. Sci. Rep..

[40] Irwin J.J., Shoichet B.K. (2005). ZINC—A free database of commercially available compounds for virtual screening. J. Chem. Inf. Model..

[41] Douguet D. (2018). Data Sets Representative of the Structures and Experimental Properties of FDA-Approved Drugs. ACS Med. Chem. Lett..

[42] (2021). Molecular Operating Environment (MOE), 2019.01.

[43] Lipinski C.A., Lombardo F., Dominy B.W., Feeney P.J. (1997). Experimental and computational approaches to estimate solubility and permeability in drug discovery and development settings. Adv. Drug Deliv. Rev..

[44] Veber D.F., Johnson S.R., Cheng H.Y., Smith B.R., Ward K.W., Kopple K.D. (2002). Molecular properties that influence the oral bioavailability of drug candidates. J. Med. Chem..

[45] Ghose A.K., Viswanadhan V.N., Wendoloski J.J. (1999). A Knowledge-Based Approach in Designing Combinatorial or Medicinal Chemistry Libraries for Drug Discovery. 1. A Qualitative and Quantitative Characterization of Known Drug Databases. J. Comb. Chem..

[46] Egan W.J., Merz K.M., Baldwin J.J. (2000). Prediction of Drug Absorption Using Multivariate Statistics. J. Med. Chem..

[47] Daina A., Michielin O., Zoete V. (2017). SwissADME: A free web tool to evaluate pharmacokinetics, drug-likeness and medicinal chemistry friendliness of small molecules. Sci. Rep..

[48] Pires D.E., Blundell T.L., Ascher D.B. (2015). pkCSM: Predicting Small-Molecule Pharmacokinetic and Toxicity Properties Using Graph-Based Signatures. J. Med. Chem..

[49] Durán Á., Zamora I., Pastor M. (2009). Suitability of GRIND-Based Principal Properties for the Description of Molecular Similarity and Ligand-Based Virtual Screening. J. Chem. Inf. Modeling.

[50] PDSP (Psychoactive Drug Screening Program) Ki Database. https://pdsp.unc.edu/databases/kidb.php.

[51] Coleman J.A., Navratna V., Antermite D., Yang D., Bull J.A., Gouaux E. (2020). Chemical and structural investigation of the paroxetine-human serotonin transporter complex. Elife.

[52] Wang S., Che T., Levit A., Shoichet B.K., Wacker D., Roth B.L. (2018). Structure of the D2 dopamine receptor bound to the atypical antipsychotic drug risperidone. Nature.

[53] Jumper J., Evans R., Pritzel A., Green T., Figurnov M., Ronneberger O., Tunyasuvunakool K., Bates R., Žídek A., Potapenko A. (2021). Highly accurate protein structure prediction with AlphaFold. Nature.

[54] Gagnon J.K., Law S.M., Brooks C.L. (2016). Flexible CDOCKER: Development and application of a pseudo-explicit structure-based docking method within CHARMM. J. Comput. Chem..

[55] Rao P.P., Pham A.T., Shakeri A., El Shatshat A., Zhao Y., Karuturi R.C., Hefny A.A. (2021). Drug repurposing: Dipeptidyl peptidase IV (DPP4) inhibitors as potential agents to treat SARS-CoV-2 (2019-nCov) infection. Pharmaceuticals.

[56] Kim S., Chen J., Cheng T., Gindulyte A., He J., He S., Li Q., Shoemaker B.A., Thiessen P.A., Yu B. (2020). PubChem in 2021: New data content and improved web interfaces. Nucleic Acids Res..

[57] Höfer P., Schosser A., Calati R., Serretti A., Massat I., Kocabas N.A., Konstantinidis A., Linotte S., Mendlewicz J., Souery D. (2013). The impact of Cytochrome P450 CYP1A2, CYP2C9, CYP2C19 and CYP2D6 genes on suicide attempt and suicide risk-a European multicentre study on treatment-resistant major depressive disorder. Eur. Arch. Psychiatry Clin. Neurosci..

[58] Pushpakom S., Iorio F., Eyers P.A., Escott K.J., Hopper S., Wells A., Doig A., Guilliams T., Latimer J., McNamee C. (2019). Drug repurposing: Progress, challenges and recommendations. Nat. Rev. Drug Discov..

[59] Cañizares-Carmenate Y., Mena-Ulecia K., MacLeod Carey D., Perera-Sardiña Y., Hernández-Rodríguez E.W., Marrero-Ponce Y., Torrens F., Castillo-Garit J.A. (2021). Machine learning approach to discovery of small molecules with potential inhibitory action against vasoactive metalloproteases. Mol. Divers..

[60] Udrescu M., Udrescu L. (2019). A Drug Repurposing Method Based on Drug-Drug Interaction Networks and Using Energy Model Layouts. Methods Mol. Biol..

[61] Udrescu L., Sbârcea L., Topîrceanu A., Iovanovici A., Kurunczi L., Bogdan P., Udrescu M. (2016). Clustering drug-drug interaction networks with energy model layouts: Community analysis and drug repurposing. Sci. Rep..

[62] Udrescu L., Bogdan P., Chiş A., Sîrbu I.O., Topîrceanu A., Văruţ >R.-M., Udrescu M. (2020). Uncovering New Drug Properties in Target-Based Drug–Drug Similarity Networks. Pharmaceutics.

[63] Sánchez-Martínez J.D., Bueno M., Alvarez-Rivera G., Tudela J., Ibañez E., Cifuentes A. (2021). In Vitro neuroprotective potential of terpenes from industrial orange juice by-products. Food Funct..

[64] Zhan Y.Y., Liang B.Q., Li X.Y., Gu E.M., Dai D.P., Cai J.P., Hu G.X. (2016). The effect of resveratrol on pharmacokinetics of aripiprazole in vivo and in vitro. Xenobiotica.

[65] Yim S.K., Kim K., Chun S., Oh T., Jung W., Jung K., Yun C.H. (2020). Screening of Human CYP1A2 and CYP3A4 Inhibitors from Seaweed In Silico and In Vitro. Mar. Drugs.

[66] Elbarbry F., Ung A., Abdelkawy K. (2018). Studying the Inhibitory Effect of Quercetin and Thymoquinone on Human Cytochrome P450 Enzyme Activities. Pharmacogn. Mag..

[67] Ganzera M., Schneider P., Stuppner H. (2006). Inhibitory effects of the essential oil of chamomile (Matricaria recutita L.) and its major constituents on human cytochrome P450 enzymes. Life Sci..

[68] Koyama S., Heinbockel T. (2020). The Effects of Essential Oils and Terpenes in Relation to Their Routes of Intake and Application. Int. J. Mol. Sci..

[69] Saiyudthong S., Mekseepralard C. (2015). Effect of Inhaling Bergamot Oil on Depression-Related Behaviors in Chronic Stressed Rats. J. Med Assoc. Thail..

[70] Garzoli S., Turchetti G., Giacomello P., Tiezzi A., Laghezza Masci V., Ovidi E. (2019). Liquid and Vapour Phase of Lavandin (Lavandula × intermedia) Essential Oil: Chemical Composition and Antimicrobial Activity. Molecules.

[71] Jamal M., Ameno K., Ameno S., Morishita J., Wang W., Kumihashi M., Ikuo U., Miki T., Ijiri I. (2007). Changes in cholinergic function in the frontal cortex and hippocampus of rat exposed to ethanol and acetaldehyde. Neuroscience.

[72] Tozar T., Santos Costa S., Udrea A.-M., Nastasa V., Couto I., Viveiros M., Pascu M.L., Romanitan M.O. (2020). Anti-staphylococcal activity and mode of action of thioridazine photoproducts. Sci. Rep..

[73] Udrea A.-M., Avram S., Nistorescu S., Pascu M.-L., Romanitan M.O. (2020). Laser irradiated phenothiazines: New potential treatment for COVID-19 explored by molecular docking. J. Photochem. Photobiol. B Biol..

[74] Avram S., Puia A., Udrea A.M., Mihailescu D., Mernea M., Dinischiotu A., Oancea F., Stiens J. (2020). Natural Compounds Therapeutic Features in Brain Disorders by Experimental, Bioinformatics and Cheminformatics Methods. Curr. Med. Chem..

